# Case Report: A case of cervical spinal epidural abscess combined with cervical paravertebral soft tissue abscess

**DOI:** 10.3389/fsurg.2022.967806

**Published:** 2022-10-05

**Authors:** Jun Cao, Jincheng Fang, Xuefei Shao, Jun Shen, Xiaochun Jiang

**Affiliations:** Department of Neurosurgery, The First Affiliated Hospital of Wannan Medical College (Yijishan Hospital of Wannan Medical College), Wuhu, China

**Keywords:** spinal epidural abscess, decompression, surgery, treatment, case report

## Abstract

**Background:**

Spinal epidural abscess (SEA) is a rare purulent infection of the central nervous system. Abscesses confined to the spinal canal can compress the spinal cord, causing nerve damage and even death in severe cases (1). Prompt diagnosis and treatment can relieve symptoms and prevent complications. To increase awareness of this rare disease, we report a case of a 58-year-old man with a cervical spinal epidural abscess combined with a soft tissue abscess in the neck and describe its clinical course, imaging feature, pathology, treatment, and patient prognosis.

**Case description:**

A 58-year-old male Chinese patient was admitted to our hospital because of neck pain for 2 months, which worsened for 4 days. On the third day of admission, the muscle strength of the limbs decreased, and MRI of the spinal cord showed abnormal signal shadows in the spinal canal at the C1-C7 level and in the surrounding soft tissue on the right side of spine, suggesting the possibility of inflammatory lesions with local abscess formation. We immediately performed decompression of the spinal canal on the patient and performed incision and drainage of the cervical abscess. During the operation, we found a large amount of pus in the epidural space of the spinal canal, and there was a fistula between the cervical abscess and the epidural abscess of the spinal canal. The patient underwent continuous drainage and anti-infective treatment with sensitive antibiotics after operation. No recurrence of the abscess was observed and the patient recovered well.

**Conclusion:**

Early diagnosis is the key to the treatment of SEA, so radiologists and neurosurgeons need to strengthen their understanding of this rare disease to avoid misdiagnosis. For SEA with definite diagnosis, decompression surgery should be performed in a timely manner when symptoms of nerve compression occur, continuous drainage should be performed after surgery, and sensitive antibiotics should be used for anti-infective treatment.

## Introduction

Spinal epidural abscess (SEA) is a rare purulent infection of the epidural space (1). Due to the accumulation of a large amount of pus and the proliferation of granulation tissue, the spinal cord is compressed and has a high disability rate. After the onset of the disease, the patient's condition develops rapidly, and a large amount of pus can accumulate in the epidural space in a short period of time, which can cause the spinal cord to be compressed, resulting in neurological dysfunction and even paraplegia (1, 2). Currently, the main treatment options for spinal epidural abscesses are sensitive antibiotic therapy or open surgery with laminectomy for decompression, removal of pus, and continuous drainage (3). Spinal epidural abscesses are more common in the thoracolumbar spinal cord and less common in the cervical spinal cord. Recently, a patient with cervical spinal epidural abscess combined with cervical soft tissue abscess was treated in our hospital, and the treatment effect was satisfactory. The report is as follows.

## Case description

**Figure 1:**
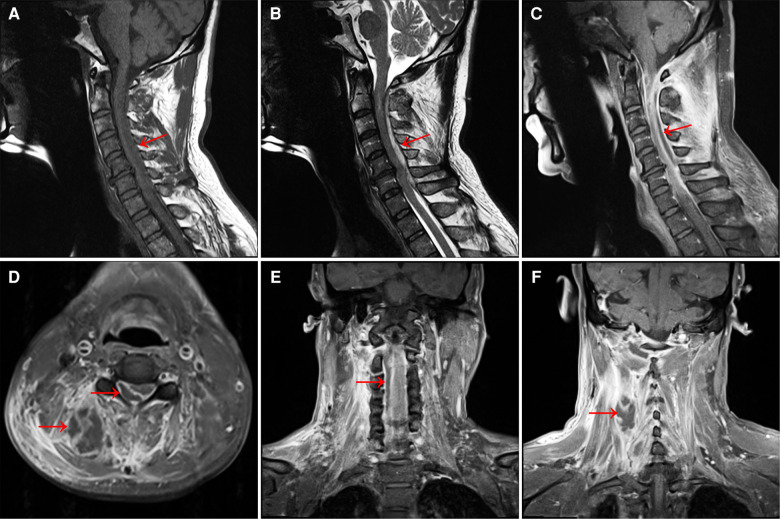
(**A**) MRI T1-weighted image shows a long strip of isointense lesions on the dorsal side of the spinal cord in the C1–C7 spinal canal. Also see C5/6 intervertebral disc herniation, and the corresponding dura and spinal cord are compressed. (**B**) T2-weighted image shows hyperintensity of the lesion. (**C**) T1-enhanced image showed annular enhancement of the lesion, no obvious internal enhancement, and spinal cord compression at the corresponding level. (**D**) Axial T1-enhanced image showed a large range of irregular non-enhanced areas in the soft tissue of the right posterior side of the cervical spine, and a ring-enhancing lesion in the right posterior part of the spinal cord in the spinal canal, with no obvious internal enhancement. (**E**) Coronal T1-enhanced image shows a slightly hypointense lesion on the right side of the spinal cord in the cervical spinal canal and (**F**) a honeycomb-like non-enhancing area in the soft tissue on the right side of the spinal canal.

The patient, a 58-year-old Chinese male, was admitted to the hospital with a 2-month history of neck pain that worsened for 4 days. In the past two months, the patient often developed neck pain without obvious cause, accompanied by radiating pain in both upper limbs and shoulders. He had been treated in a local hospital and was considered as having “cervical spondylosis”. 14 days before admission, he received neck acupuncture treatment at the local hospital, but the neck pain did not improve significantly. 4 days before admission, she developed redness, swelling and pain in her neck, and fever appeared. The highest temperature reached 38.6°C in the afternoon. After admission, a neck B-ultrasound showed an abnormal echo mass in the deep soft tissue of the right posterior neck. On the third day of admission, the muscle strength of the limbs decreased, and the decrease of the muscle strength of the lower limbs was particularly obvious. Physical examination showed extensive percussion pain in the spinous process of the neck with radiating pain in both upper limbs. The left biceps muscle strength was grade 3, the right biceps muscle strength was grade 2, the quadriceps muscle strength of both lower limbs was grade 2, the left foot dorsiflexor muscle strength was grade 2, the right foot dorsiflexor muscle strength was grade 0, and the anal sphincter muscle strength was grade 0. MRI of the cervical and thoracic vertebrae was immediately performed, showing abnormal signal shadow of dorsal spinal cord in the spinal canal at C1–C7 level, inflammatory lesions with the possibility of local abscess formation, posterior herniation of the C5/6 disc, and compression of the surrounding dura and spinal cord (Figure 1). The patient had a history of hypertension for 7 years, with normal blood pressure control, and denied the history of diabetes and other diseases.

**Figure 2:**
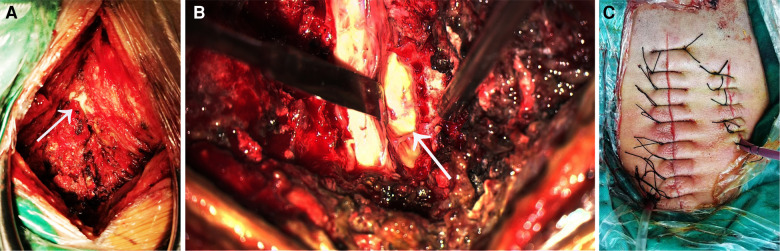
(**A**) during the operation, a fistula was seen in the right posterior side of the spinal canal, connecting the paravertebral abscess and the spinal epidural abscess.The arrow points to the location of the fistula. (**B**) After opening the lamina, a large amount of pus was seen in the spinal canal, and the pus was mainly located in the back of the spinal cord and the right side of the spinal cord. The arrow points to the pus on the side of the spinal cord. (**C**) The surgical incision and drainage tube for the spinal abscess are shown on the left, and the surgical incision and drainage tube for the paravertebral soft tissue abscess in the neck are shown on the left.

Considering that the patient had developed neurological symptoms and was at risk of respiratory failure at any time due to cervical spinal cord compression, the patient was immediately treated with “cervical spinal epidural abscess removal + spinal canal decompression + neck abscess incision and drainage” under general anesthesia. The C4 spinous process was used as the center to mark a straight incision with a length of 8 cm in the posterior midline of the neck. The incision was made layer by layer, and the bilateral paraspinal muscles were dissected to the lateral edge of the lamina under the periosteum. During the operation, an abscess fistula was seen in the right paravertebral muscle, connecting the soft tissue abscess of the neck to the spinal canal. A large amount of dark yellow epidural pus spilled when the C3–C5 lamina was opened (Figure 2). The pus was collected for bacterial culture and drug sensitivity test. Part of ligamentum flavum was resected and sent for pathological examination. During the operation to remove the pus in the spinal canal, it is necessary to decompress the spinal canal thoroughly, but also to protect the dura mater to prevent pus from breaking into the subarachnoid space and causing subarachnoid space infection. After complete decompression, the spinal canal was rinsed with iodophor solution, hydrogen peroxide and a large amount of normal saline, and the spinal cord was found to be pulsating well without obvious spinal cord compression. One negative pressure drainage tube was indwelled and the incision was closed successively. Further incision and drainage were performed for the abscess in the deep neck. A vertical incision of 4.5 cm was made about 3 cm beside the midline of the posterior neck. The skin and subcutaneous tissue were cut layer by layer, and the abscess cavity was observed in the depth of the neck. The pus in the cavity was sucked out, and a negative pressure drainage tube was placed and sutured successively to close the incision.

**Figure 3:**
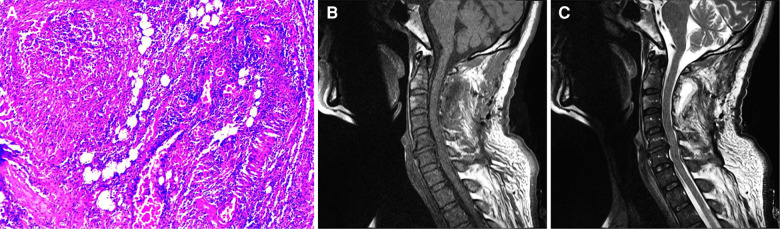
(**A**) microscopic examination of the sample tissue with HE staining revealed fibrous adipose tissue, in which a large number of acute and chronic inflammatory cell infiltrates with abscess formation, and vascular dilatation and hyperemia were observed. Picture was taken at 100 magnification. (**B**) T1-weighted image of the cervical spine 21 days after the operation showed that the abscess signal in the spinal canal disappeared. (**C**) T2-weighted image of the cervical spine 21 days after the operation.

After surgery, the patient was transferred to neurosurgical intensive care unit for observation, assisted breathing by ventilator, anti-inflammatory treatment was performed by intravenous infusion of vancomycin, symptomatic treatment was performed by nutritive nerve drugs, and continuous drainage was performed by negative pressure drainage tube. On day 2 after surgery, the muscle strength of the patient's left biceps muscle was grade 4, the muscle strength of the right biceps muscle was grade 3, the muscle strength of the quadriceps femoris muscle of both lower limbs was grade 4, and the muscle strength of the anal sphincter muscle was grade 4. The patient was able to control urination and remove the catheter 5 days after surgery. Bacterial culture and drug sensitivity test showed Staphylococcus aureus (susceptible to vancomycin, levofloxacin and ceftriaxone), and pathological results showed inflammatory reactions (Figure 3). Drainage tube was removed 10 days after surgery and vancomycin was replaced with ceftriaxone (continued for 5 weeks). Twenty days after surgery, the patient got out of bed with a neck brace and moved autonomously. MRI of the cervical spine 21 days after the operation showed that the abscess signal in the spinal canal disappeared completely (Figure 3). After 6 months of follow-up, the patient had grade 5 muscle strength, no sensory disturbance, and no recurrence of abscess.

## Discussion

The infection of spinal epidural abscess is mainly through the following three ways (3): (1) Blood and lymphatic system infection; (2) Direct spread of local infection; (3) Invasive surgical procedures such as the placement of epidural catheter, result in bacterial invasion of the spinal canal. Among them, the placement of epidural catheter is an important risk factor for SEA. Some studies (4, 5) reported that the incidence of epidural abscess after the placement of epidural catheter is 0.5%∼3.0%. Other possible risk factors include diabetes, human immunodeficiency virus (HIV) infection, trauma, tattooing, acupuncture, and infection of adjacent bone or soft tissue. In this case, combined with the intraoperative findings, the infection route was considered to be the spread of the cervical paravertebral soft tissue abscess into the spinal canal. This patient denied the history of diabetes mellitus and other diseases, but had received neck acupuncture treatment before the onset of the disease. It cannot be ruled out that acupuncture treatment was the cause of the disease.

The common pathogenic bacteria of SEA are Staphylococcus aureus, while Escherichia coli and Streptococcus pneumoniae are rare (6–8). The mechanism of spinal cord injury caused by abscess diffusion in epidural space is as follows (3): (1) Direct compression of spinal cord; (2) Thrombosis and thrombophlebitis of adjacent veins; (3) Interruption of arterial blood supply; (4) Bacterial toxins and inflammatory mediators. When the infection occurs in the epidural space, the abscess can spread longitudinally to 3–5 spinal segments, and in severe cases it can spread to the entire spine (9). However, even small SEA can cause severe neurological symptoms and sequelae, and neurological impairment is the most serious complication of spinal epidural abscess (10).

SEA is more common in the thoracolumbar spine, an area where the epidural space is larger and contains more fatty tissue that is susceptible to infection (10). Most of the SEA is located in the posterior part of the spinal canal, and if it occurs in the anterior part, it is usually located below L1. The epidural space is a vertical sheath-like structure, and an abscess that begins at one segment often expands to multiple segments (3). Our case is unique in that the SEA occurred at almost the entire cervical vertebra segment, forming neurological symptoms with obvious spinal cord compression.

The initial manifestations of SEA are usually non-specific, and the classical triad with diagnostic significance is fever, spinal pain and neurological dysfunction (11). However, only a few patients have all three manifestations at the time of diagnosis. When the patient has severe localized neck and back pain and fever, the diagnosis can be considered as SEA or spinal osteomyelitis. However, when a patient seeks a doctor only for neck pain, SEA is rarely considered, and it is easy to miss the diagnosis. When SEA is combined with paravertebral abscess, it can also manifest as inflammatory manifestations of redness, swelling, heat, and pain in the paravertebral soft tissue.

Once SEA is suspected, imaging should be performed as soon as possible. MRI examination can show the location and extent of intraspinal lesions, and can even show fistulas between abscesses, which is the preferred examination method (12). In the acute phase, SEA manifests as low signal intensity or iso-intensity on T1WI and high signal intensity on T2WI. As the disease progresses, it will show iso-intensity on T1WI and T2WI, and the abscess wall will be significantly enhanced on enhanced scan, but there is no enhancement in the abscess cavity. MRI can help differentiate SEA from intraspinal hematoma and spinal epidural tumor. Intraspinal hematoma is mainly iso-intensity and highintensity on T1WI, and slightly high-intensity or high-intensity on T2WI, mixed with a little low-intensity. Enhanced scan shows the enhancement of the hematoma wall. Epidural tumors show isointensity on T1WI, slightly hyperintensity or isointensity on T2WI, and obvious homogeneous enhancement on enhanced scan (13). Contrast-enhanced CT scans can be used if MRI cannot be performed immediately or if there are contraindications (14). X-ray examination has little diagnostic value for SEA, and myelography has been basically eliminated.

In recent years, there have been many debates on how to treat spinal epidural abscesses. Some scholars advocate the use of sensitive antibiotics alone, while others believe that open surgery assisted by sensitive antibiotics is more reliable. Suppiah et al. (15) believed that open surgery assisted with sensitive antibiotic therapy is the best treatment method. If the neurological function is intact, conservative antibiotic treatment can be given first, but close neurological function monitoring is required. Patients with suspected spinal epidural abscess should be closely monitored for neurological function. Tuchman et al. (16) believed that patients should be clear about delayed neurological damage that may occur at any time when they are treated with antibiotics alone for SEA patients without neurological symptoms, but because the irreversible time point of spinal nerve damage is still unclear, the first recommended treatment is surgical treatment. Shweikeh et al. (17) regard the occurrence of nerve damage as the key factor for surgical treatment, and the timing of surgical treatment should be within 36∼72 h after the occurrence of nerve dysfunction. The timing of surgery should also be determined by the patient's age, complications (diabetes, cardiovascular disease, renal insufficiency) and other specific conditions (18).

In this case, MRI examination was performed immediately after the onset of neurological symptoms and decompression of the spinal canal was performed in time, which is the key to the good prognosis of the patient. After surgery, vancomycin was used empirically for anti-inflammatory treatment. The postoperative drug sensitivity test showed that the bacteria were sensitive to vancomycin, levofloxacin and ceftriaxone. After the drainage tube was removed, vancomycin was replaced with ceftriaxone to continue treatment for 5 weeks. Follow-up MRI re-examination showed that the epidural abscess disappeared and the compression was relieved. In this case, only spinal canal decompression was performed, and internal fixators were not used. The main considerations were as follows: (1) The pathogenic bacteria were not clear, and the use of internal fixators might increase the risk of infection; (2) No stability structures such as articular processes were destroyed during operation, and postoperative spinal stability was not affected.

## Conclusion

Early diagnosis is the key to the treatment of SEA. Patients without neurological symptoms can be treated with effective antibiotics in early, sufficient and timely manner. If neurological symptoms worsen, MRI examination should be performed in time. For SEA with definite diagnosis, decompression surgery should be performed in time when symptoms of nerve compression occur, and continuous drainage and anti-infection treatment with sensitive antibiotics should be selected after surgery.

## Data Availability

The original contributions presented in the study are included in the article/Supplementary Material, further inquiries can be directed to the corresponding author/s.

## References

[1] Zimmerer SM Conen A Müller AA Sailer M Taub E Flückiger U Spinal epiduralabscess: aetiology, predisponent factors and clinical outcomes in a 4-year prospective study. Eur Spine J. (2011) 20(12):2228–34. 10.1007/s00586-011-1838-y 21590496 PMC3229722

[2] Epstein NE. Timing and prognosis of surgery for spinal epidural abscess: a review. Surg Neurol Int. (2015) 6(Suppl 19):S475–486. 10.4103/2152-7806.166887 26605109 PMC4617026

[3] Babic M Simpfendorfer CS Berbari EF. Update on spinal epidural abscess. Curr Opin Infect Dis. (2019) 32:265–71. 10.1097/QCO.0000000000000544 31021957

[4] Cook TM Counsell D Wildsmith JA, Royal College of Anaesthetists Third National Audit Project. Major complications of central neuraxial block: report on the Third National Audit Project of the Royal College of Anaesthetists. Br J Anaesth. (2009) 102(2):179–90. 10.1093/bja/aen360 19139027

[5] Sethna NF Clendenin D Athiraman U Solodiuk J Rodriguez DP Zurakowski D. Incidence of epidural catheter-associated infections after continuous epidural analgesia in children. Anesthesiology. (2010) 113(1):224–32. 10.1097/ALN.0b013e3181de6cc5 20526185

[6] Sadato N Numaguchi Y Rigamonti D Kodama T Nussbaum E Sato S Spinal epidural abscess with gadolinium-enhanced MRI: serial follow-up studies and clinical correlations. Neuroradiology. (1994) 36(1):44–8. 10.1007/BF00599195 8107997

[7] Hadjipavlou AG Mader JT Necessary JT Muffoletto AJ. Hematogenous pyogenic spinal infections and their surgical management. Spine. (2000) 25(13):1668–79. 10.1097/00007632-200007010-00010 10870142

[8] Felton B Dao T Gerstner B Letarte S. Diagnosis of spinal epidural abscess by abdominal plain - film radiography. West J Emerg Med. (2014) 15(7):885–6. 10.5811/westjem.2014.8.22545 25493143 PMC4251244

[9] Darouiche RO Hamill RJ Greenberg SB Weathers SW Musher DM. Bacterial spinal epidural abscess. Review of 43 cases and literature survey. Medicine (Baltimore). (1992) 71(6):369–85. 10.1097/00005792-199211000-00004 1359381

[10] Vakili M Crum-Cianflone NF. Spinal epidural abscess: a series of 101 cases. Am J Med. (2017) 130(12):1458–63. 10.1016/j.amjmed.2017.07.017 28797646

[11] Davis DP Wold RM Patel RJ Tran AJ Tokhi RN Chan TC The clinical presentation and impact of diagnostic delays on emergency department patients with spinal epidural abscess. J Emerg Med. (2004) 26(3):285–91. 10.1016/j.jemermed.2003.11.013 15028325

[12] Tompkins M Panuncialman I Lucas P Palumbo M. Spinal epidural abscess. J Emerg Med. (2010) 39(3):384–90. 10.1016/j.jemermed.2009.11.001 20060254

[13] Uchida K Nakajima H Yayama T Sato R Kobayashi S Chen KB Epidural abscess associated with pyogenic spondylodiscitis of the lumbar spine; evaluation of a new MRI staging classification and imaging findings as indicators of surgical management: a retrospective study of 37 patients. Arch Orthop Trauma Surg. (2010) 130(1):111–8. 10.1007/s00402-009-0928-3 19565251

[14] Johnson KG. Spinal epidural abscess. Crit Care Nurs Clin North Am. (2013) 25(3):389–97. 10.1016/j.ccell.2013.04.002 23981455

[15] Suppiah S Meng Y Fehlings MG Massicotte EM Yee A Shamji MF. How best to manage the spinal epidural abscess? A current systematic review. World Neurosurg. (2016) 93:20–8. 10.1016/j.wneu.2016.05.074 27262655

[16] Tuchman A Pham M Hsieh PC. The indications and timing for operative management of spinal epidural abscess: literature review and treatment algorithm. Neurosurg Focus. (2014) 37(2):E8. 10.3171/2014.6.FOCUS14261 25081968

[17] Shweikeh F Saeed K Bukavina L Zyck S Drazin D Steinmetz MP. An institutional series and contemporary review of bacterial spinal epidural abscess: current status and future directions. Neurosurg Focus. (2014) 37(2):E9. 10.3171/2014.6.FOCUS14146 25081969

[18] Ran B Chen X Zhong Q Fu M Wei J. CT-guided minimally invasive treatment for an extensive spinal epidural abscess: a case report and literature review. Eur Spine J. (2018) 27(Suppl 3):380–5. 10.1007/s00586-017-5307-0 28951964

